# The Association of Iron and the Pathologies of Parkinson’s Diseases in MPTP/MPP^+^-Induced Neuronal Degeneration in Non-human Primates and in Cell Culture

**DOI:** 10.3389/fnagi.2019.00215

**Published:** 2019-08-30

**Authors:** Liangqin Shi, Chao Huang, Qihui Luo, Edmond Rogers, Yu Xia, Wentao Liu, Wenjing Ma, Wen Zeng, Li Gong, Jing Fang, Li Tang, Anchun Cheng, Riyi Shi, Zhengli Chen

**Affiliations:** ^1^Laboratory of Animal Disease Model, College of Veterinary Medicine, Sichuan Agricultural University, Chengdu, China; ^2^Department of Basic Medical Sciences, College of Veterinary Medicine, Purdue University, West Lafayette, IN, United States; ^3^Sichuan Primed Shines Bio-Tech Co., Ltd./National Experimental Macaque Reproduce Laboratory, Chengdu, China; ^4^Key Laboratory of Animal Disease and Human Health of Sichuan Province, College of Veterinary Medicine, Sichuan Agricultural University, Chengdu, China

**Keywords:** Parkinson’s disease, iron, lipofuscin, oxidative stress, neuronal degeneration, rhesus monkey

## Abstract

Despite much efforts in the last few decades, the mechanism of degeneration of dopamine (DA) neurons in the substantia nigra (SN) in Parkinson’s disease (PD) remains unclear. This represents a major knowledge gap in idiopathic and genetic forms of PD. Among various possible key factors postulated, iron metabolism has been widely suggested to be involved with fueling oxidative stress, a known factor in the pathogenesis of PD. However, the correlation between iron and DA neuron loss, specifically in the SN, has not been described in experimental animal models with great detail, with most studies utilizing rodents and, rarely, non-human primates. In the present study, aiming to gain further evidence of a pathological role of iron in PD, we have examined the correlation of iron with DA neuron loss in a non-human primate model of PD induced by MPTP. We report a significant iron accumulation accompanied by both DA degeneration in the SN and motor deficits in the monkey that displayed the most severe PD pathology and behavioral deficits. The other two monkeys subjected to MPTP displayed less severe PD pathologies and motor deficits, however, their SN iron levels were significantly lower than controls. These findings suggest that high iron may indicate and contribute to heightened MPP^+^-induced PD pathology in late or severe stages of PD, while depressed levels of iron may signal an early stage of disease. Similarly, using a cell culture preparation, we have found that high doses of ferric ammonium citrate (FAC), a factor known to enhance iron accumulation, increased MPP^+^-induced cell death in U251 and SH-SY5Y cells, and even in control cells. However, at low dose FAC restored or increased the viability of U251 and SH-SY5Y cells in the absence or presence of MPP^+^. These observations imply that high levels of iron likely contribute to or heighten MPP^+^ toxicity in the later stages of PD. While we report reduced iron levels in the earlier stages of MPTP induced PD, the significance of these changes remains to be determined.

## Introduction

Parkinson’s disease (PD) is a well-known progressive neurodegenerative disorder that involves significant degeneration of dopamine (DA) neurons in the substantia nigra (SN) (Riederer and Wuketich, 1976; Agid, 1991). Such cellular neurodegeneration is accompanied by progressive, and usually irreversible motor deficits, marked by bradykinesia, rigidity, and tremor (Foley and Riederer, 2000). Although the mechanism of DA cell death in PD is far from clear, oxidative stress is considered to play a major role.

Iron, a redox-active transition metal, is a critical player in many physiological processes in the nervous system, such as myelin synthesis, DA metabolism, and neurotransmitter regulation (Zecca et al., 2004; Moos et al., 2007; Todorich et al., 2009). However, excess iron can intensify oxidative stress and increase reactive oxygen species (ROS), resulting in damage to DNA, RNA, proteins and lipids (Zecca et al., 2004; Moos et al., 2007; Todorich et al., 2009). Current evidence suggests that iron is selectively elevated in the SN of PD patients (Dexter et al., 1989; Vymazal et al., 1999; You et al., 2015). Further, the degree of iron accumulation has been shown to be correlated to disease severity in human PD (Martin-Bastida et al., 2017; An et al., 2018). This has been corroborated in animal models where unilateral injection of iron to the SN of rats resulted in DA neuron degeneration (Ben-Shachar and Youdim, 1991), infusion of ferric chloride into the SN of rats resulted in a dose-dependent progression of parkinsonism (Sengstock et al., 1993), and 1-methyl-4-phenyl-1,2,3,6-tetrahydropyridine (MPTP) injection into the brain led to iron accumulation in the SN of monkeys (Mochizuki et al., 1994; He et al., 2003). However, human studies do not allow for the type of interrogative resolution necessary to uncover the role of iron accumulation in PD pathogenesis. In addition, related animal studies are primarily rodent-based and fail to fully replicate the human disease pathology.

Non-human primate models of neurodegenerative diseases, particularly related to PD, are very effective methods of investigation due to their close relationship with humans. In addition, very few non-human primate studies have begun to investigate this phenomenon. Therefore, the primary goal of this study was to further document the correlation of iron with oxidative stress and DA cell death in a monkey model of MPTP-induced PD. A parallel study using cell culture aimed to investigate the direct causality of iron accumulation and the death of a human glial and neuronal cell line in the presence of MPP^+^. Utilizing this combination strategy, we have demonstrated in a limited number of monkeys (*n* = 3) that the highest level of SN iron accumulation correlated with the following: highest loss of DA neurons, greatest oxidative stress in the SN, and the most severe PD-like motor deficits. Furthermore, the monkeys that exhibited less severe neuronal pathology and motor deficits had lower SN iron levels than control monkeys, further implying a correlation between iron and PD pathology. Parallel *in vitro* studies showed that high doses of ferric ammonium citrate (FAC), which is known to enhance iron accumulation, increased cell death in U251 and SH-SY5Y cells with or without MPP^+^, while low levels of FAC did not exacerbate cell death (under the same conditions).

## Materials and Methods

### Animals and MPTP Treatment

All the experimental protocols were reviewed and approved by the Animal Welfare and Use Committee, and all the experimental procedures were in conformity with the guidance of the National Institutes of Health Guide for the Care and Use of Laboratory Animals of the United States. In total, six healthy female rhesus monkeys (aged 13–16 years, and weighed 5.4–7.0 kg at the start of the study) were obtained from Sichuan Primed Biological Technology Co., Ltd (National Experimental Macaque Reproduce Laboratory) (Certificate No SCXK (Chuan): 2013-105). Two weeks prior to the experiment, the animals were transferred from their home room to stainless steel monkey cages, one animal per cage, in a feeding room with controlled conditions of temperature (19 to 26°C), humidity (40 to 70%) and light (12 h day and night cycles, lights on 8:00 am). Tap water was provided *ad libitum* via an automatic bubbler. A standard diet (containing 18% protein, 69% carbohydrates, 3% fat, and 10% water) was fed twice daily. Meanwhile, vegetables and fruits with equal nutrients were provided to each animal every day. The healthcare and maintenance of non-human primates was performed under the supervision of specialty veterinarians. All biohazard waste was autoclaved before disposal.

**FIGURE 1:**
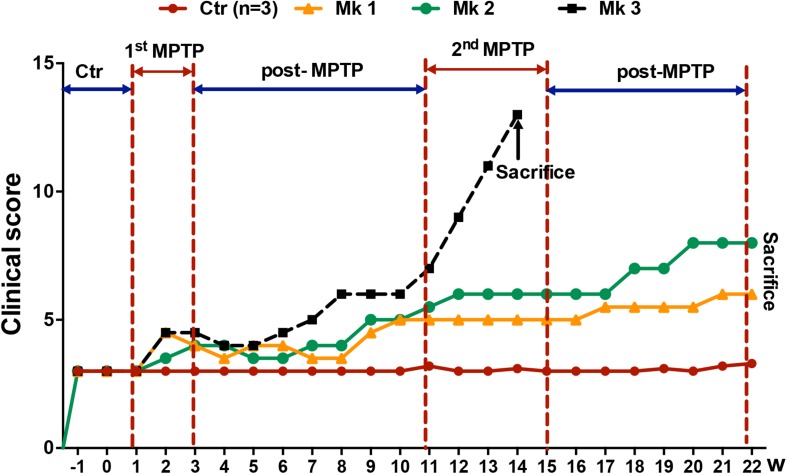
Experimental design and clinical behavioral scores of MPTP or saline-injected monkeys. The evaluation of clinical behavioral deficits was expressed as the clinical scores which were determined weekly for up to a total of 22 weeks. Higher scores indicate more severe clinical deficits. Overall, there were a total six monkeys, three MPTP-injected and three saline-injected. The clinical score was determined for 2 weeks before injection of MPTP or saline. Note that there were two periods of MPTP injection, the first lasting for 2 weeks (1^st^ MPTP) and second for 4 weeks (2^nd^ MPTP). Both MPTP-injection periods were followed by an interval of no injection, 8 and 7 weeks. Note that the clinical score increased conspicuously starting at the beginning of 2nd MPTP injection period. Interestingly, only Mk3 showed classical clinical characteristics of PD. Unfortunately, the PD symptoms of Mk3 worsened rapidly to a level that was too severe to continue. Therefore, Mk3 was sacrificed prematurely at week 14 based on humanitarian considerations. Mk1 and Mk2, on the other hand, only developed mild PD symptoms (mild bradykinesia) and were able to continue to be observed for the entire length of study (22 weeks).

The monkeys were randomly divided into two groups: normal (control) group (*n* = 3) and MPTP group (*n* = 3). The MPTP group and normal group were administered with a small dose (0.2 mg/kg) of either 1-methyl-4-phenyl-1,2,3,6-tetrahydropyridine (MPTP-HCL, Sigma, St. Louis, MO, United States) or the same dose of saline, respectively, by intramuscular injection daily for 45 days (detailed protocol see Table 1 and Figure 1). Specifically, there were two periods of MPTP injection, the first lasting for 15 days (1^st^ MPTP) and the second for 30 days (2^nd^ MPTP). Both MPTP-injection periods were followed by an interval of no injection, 8 and 7 weeks, respectively.

**TABLE 1:** Information of animals and MPTP treatment.

	**Weigh**	**Age**		**Injection**	**Single**	**Injection**	**Total dose of**
**Animal**	**(Kg)**	**(Year)**	**Method**	**reagent**	**dose**	**times**	**MPTP (mg/kg)**
Mk 1	5.4	14	Intramuscular injection	MPTP	0.2 mg/kg	37	7.4
Mk 2	5.7	13		MPTP	0.2 mg/kg	45	9.0
Mk 3	7	13		MPTP	0.2 mg/kg	45	9.0
Control group (*n* = 3)	5.8 ± 0.5	14 ± 0.6		Saline	0.5 ml/kg	45	0

### Clinical Motor Evaluation

Monkeys were monitored by video surveillance with no observers in the room for recording the spontaneous activity during all the procedures. Clinical scoring was analyzed by three examiners familiar with the scoring rules modified from previous studies (van der Stelt et al., 2005; Fox et al., 2012). Briefly, motor function including movement (0–9), posture (0–1), and bradykinesia (0–3) was assessed, and the scores represented maximal (range of movement) or typical (posture, bradykinesia) behavior in the defined time period. Increasing levels of parkinsonism were reflected by an increased range of the scores. The spontaneous activity was assessed by the average score of the week.

### Tissue Collection

Animals were sacrificed under deep anesthesia by sodium pentobarbital overdose (50 mg/kg, i.v, with effect confirmed by absence of corneal reflex), and the brains were rapidly removed on ice, divided into coronal slabs of 4 mm thickness and then fixed in 4% paraformaldehyde phosphate buffer solution (pH 7.4) for further use. Brain slabs were then cryoprotected by soaking in 20 and 30% sucrose solution at 4°C and sliced into 30 μM coronal sections.

### Histopathology

The sections were floated in 0.1% chrome alum-gelatin solution and mounted in slides. After drying at room temperature, the sections were processed for hematoxylin and eosin (H&E) staining, Nissl staining (Glaser and Van der Loos, 1981), Prussian blue staining (Sundberg and Broman, 1955; Sharma and Nehru, 2018) and lipofuscin (Schmorl) staining (Goldfischer and Bernstein, 1969).

### Immunohistochemical Analysis

Sections for immunohistochemistry were rinsed thrice in 0.01 M PBS (pH = 7.4) and then placed in hydrogen peroxide [V(30% H_2_O_2_)_:_V(methanol) = 1: 50] to eliminate endogenous peroxidase activity. After washing three times in PBS, sections were incubated in 10% normal goat serum for 30 min at 37°C, and then sections were reacted with primary antibodies (TH: rabbit anti-TH polyclonal, 1:200, ENZO) for 24 h at 4°C. After several washing steps, sections were incubated with biotinylated secondary antibody followed with washing steps and incubation with streptavidin peroxidase for 1 h at room temperature. Finally, the sections were stained in the ammonium nickel sulfate-DAB for no more than 30 min. The staining solution was prepared as described previously (Chen et al., 2007).

### Subdivisions of the SN

The anatomical position of the brain nuclei was identified according to the Macaque Brain Atlas (Szabo and Cowan, 1984; Yang et al., 1990). To decipher the impact of MPTP-induced neuronal loss and regional vulnerability within the SN, we divided the SN into seven tiers. Briefly, according to this stereotaxic method, two coordinate values on the same drawing of coronal sections were marked, one shows the coordinates value of the distance from APO plane and the other shows the value of the distance from the posterior margin of C.P. Then, the SN was divided into 7 tiers: CP + 8.4/A15.2, CP + 7.2/A14.0, CP + 6.6/A13.4, CP + 5.9/12.7, CP + 4.8/A11.6, CP + 3.7/A10.5, CP + 1.6/A8.4 (Supplementary Figure S1). Meanwhile, the nigral tiers of CP + 6.6/A13.4 and CP + 5.9/12.7 were divided into three subregions: ventral, medial and dorsal (Supplementary Figure S1).

### Image Analysis and Data Processing

Tissue sections were observed by a light microscopy (Nikon, Tokyo, Japan), and images were captured using a Nikon50i-BF fluorescent biological digital microscope (Nikon) equipped with a CCD camera (Nikon). The region of interest (ROI) was outlined under low magnification (4×) and then captured in high magnification (40×–400×). For measurement of DA neuronal size by Nissl staining, the ROI images were captured at high magnification (400×) and then the long and minor axis sizes were measured using Image-Pro Plus 6.0 according to the scale bar. For estimation of TH(+) positive areas and staining areas of iron in seven nigral tiers; three or four intact sections in each tier were selected for analysis and then sequential ROIs were captured and quantified using Image-Pro Plus 6.0. For estimation of lipofuscin cells, the ROI images were also captured at high magnification (200×) and quantified using Image-Pro Plus 6.0.

### Cell Culture

U251 and SH-SY5Y cells were used in this study (both purchased from Kunming Cell Bank of Chinese Academy of Sciences, China) as they have been used in previous, similar studies (Tanji et al., 2001; Deng et al., 2013; Xicoy et al., 2017). Both cell lines were routinely cultured in DMEM (Gibco, Waltham, MA, United States) supplemented with 10% FBS and 1% penicillin/streptomycin and maintained at 37°C in a humidified atmosphere of 5% CO_2_. Twenty-four hours after plating or when the cell density was 60–70%, the cells were immediately treated with ferric ammonium citrate (FAC) for 24 h or pretreated with FAC for 12 h followed by MPP^+^ (Sigma, United States) for another 12 h.

### Cell Viability Assay

Both U251 and SH-SY5Y cells were plated in 96-well plates, respectively. After treatment, cell viability was measured by a Cell Counting Kit-8 (CCK-8) system according to the instructions provided by the manufacturer (Dojindo, CK04-11, Japan). Briefly, CCK-8 solution (10 μL per 100 μL of medium in each well) was added in each well, and the plates were then incubated at 37°C for 1 h. The absorbance of each well was read at 450 nm using a microplate (Thermo, United States) reader. Each experiment was repeated four times.

### Statistical Analysis

Statistical analysis was carried out using IBM SPSS Statistics 22. Data are expressed as mean ± SD, and one-way analysis of variance (ANOVA) with Duncan’s multiple range test and Student’s *t*-test were used for statistical assessment. *P* < 0.05 was considered statistically significant.

## Results

### Clinical Motor Function Evaluation of Control and MPTP-Treated Monkeys

Behavior tests revealed no apparent differences of clinical symptoms in MPTP-treated monkeys after the first 15 daily injections of MPTP (1^st^ MPTP, Figure 1) when compared with the control group. However, the behavioral scores exhibited a progressive trend of deterioration during the first post-MPTP period (8 weeks), while the trend was accelerated toward the end of this period (Figure 1). Subsequently, the second injection of MPTP (2^nd^ MPTP, Figure 1) was carried out which lasted for 4 weeks. During this period, classical characteristics of PD, including resting tremor, rigidity, bradykinesia, and postural instability were observed and worsened rapidly in Monkey 3 (Mk3) after the 21st injection in the 2nd MPTP period. In addition, feeding for Mk3 was also severely impeded. For humanitarian and animal welfare considerations, Mk3 was sacrificed after deep anesthesia. Meanwhile, the remaining 2 MPTP-treated monkeys (Mk1 and Mk2) displayed mild bradykinesia without tremor during 2nd MPTP and 2nd post-MPTP periods (Figure 1 and Table 1). As such, Mk1 and Mk2 were observed for the entire experimental period and sacrificed at the end of 22 weeks of evaluation. In addition, all the control animals were sacrificed at the same time (at the end of 22 weeks of evaluation).

### MPTP Injection Produced DA Neuron Atrophy and Cell Loss

**FIGURE 2:**
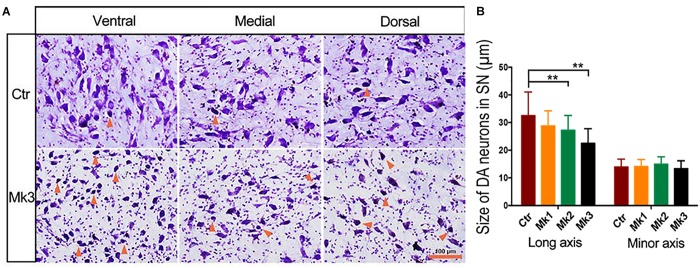
Photomicrographs of Nissl staining in the SNc in a control and in a MPTP-injected monkey. **(A)** The micrograph of Nissl staining in control and in monkey 3 (Mk3) that displayed the most severe structural and functional deficits. The images were taken from ventral, medial, and dorsal areas of the substantia nigra (SN) for both monkeys. Note that in control monkeys, only a few atrophic neurons (with darker color and smaller soma size than normal neurons, arrows) were seen. In addition, neurites can be clearly observed. In Mk3, however, more atrophic neurons can be discerned, and the neurites were less abundant than the control, especially in ventral SN. **(B)** Quantification of the size of Nissl -stained neurons in control and in three MPTP-injected monkeys, Mk1, Mk2, and Mk3. The value of control represents the average of three control monkeys. Note the significant decrease of the size of Nissl -stained neurons in Mk2 [*F*(3,122) = 8.607, *P* < 0.0001] and Mk3 [*F*(3,141) = 5.579, *P* = 0.0012] compared to control, when measurement was based on long axis, but not when based on minor axis. ANOVA, ^∗∗^*P* < 0.01 and Mk3.

**FIGURE 3:**
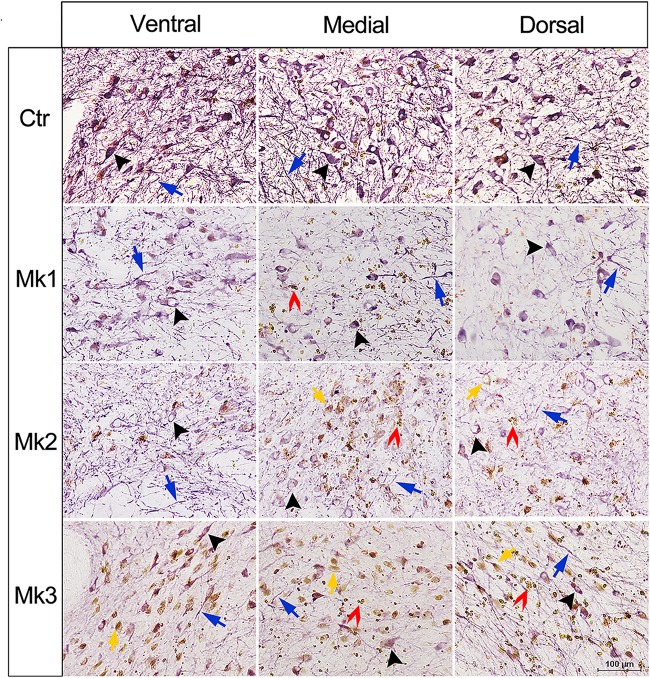
Micrograph of immunohistostaining of TH^+^ neurons in the SN regions of control and MPTP-injected monkeys. Immunohistostaining of Tyrosine hydroxylase (TH) in the SNc in control and MPTP-lesioned monkeys. The images were taken from ventral, medial, and dorsal area of the SN for all involved monkeys. Black arrows indicate the neuronal soma and blue arrows denote neurites with positive TH (+) staining. In MPTP-lesioned monkeys, both neuronal somas and neurites were damaged, and the damage in Mk3 is more obvious than Mk2 and Mk1. In addition, brown dots were detected in soma (yellow arrows and inset) and in the extracellular (red arrows and inset).

**FIGURE 4:**
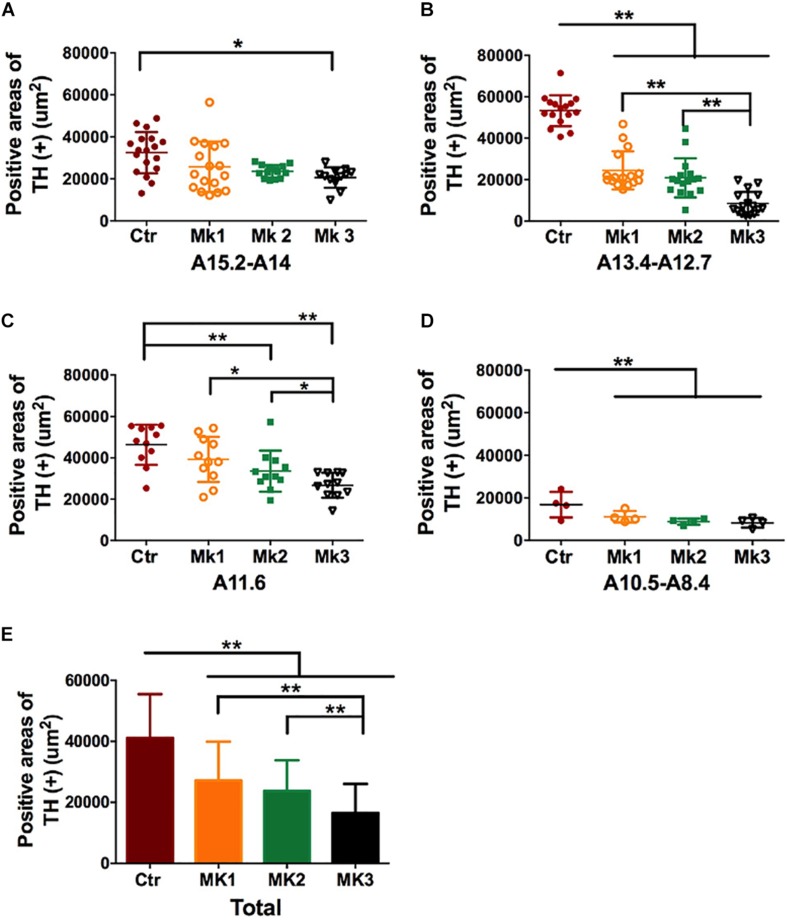
Determination and comparison of TH staining in the SN in three MPTP-lesioned monkeys and in controlled monkeys. The data of TH (+) staining was collected, and stereology analysis was carried out in four SN tiers for each monkey, A15.2-A14 **(A)**, A13.4-A12.7 **(B)**, A11.6 **(C)**, and A10.5-A8.4 **(D)** Note that TH (+) staining was consistently lower in the Mk3 compared to controls in all tiers [*F*(3,186) = 33.42, *P* < 0.0001]. In addition, TH (+) staining in Mk3 was also significantly lower than Mk1 and 2 in tier A13.4-A12.7 [*F*(2,45) = 16.46, *P* < 0.0001] and A11.6 [*F*(2,30) = 5.075, *P* = 0.0126]. **(E)** A comparison of TH (+) area in each monkey when the data were pooled together from all the SN tiers. Analysis showed that TH (+) positive areas in the SN of all three MPTP-lesioned monkeys were significantly reduced when compared to control [*F*(3,279) = 43.5, *P* < 0.0001]. In addition, TH (+) areas in Mk3 were significantly lowered than that of Mk1 and Mk2[*F*(2,133) = 11.14, *P* < 0.0001]. ANOVA, ^∗^*P* < 0.05, ^∗∗^*P* < 0.01.

Histological examination revealed no damage to the liver and kidney of the 3 MPTP-injected monkeys (data not shown). Tissue analysis using Nissl staining showed that DA neurons in the SN of MPTP-injected monkeys were atrophied, especially in Mk3 (Figure 2A). Quantitative stereology demonstrated a significant decrease in neuron size (long axis) of Mk2 [*F*(3,122) = 8.607, *P* < 0.0001] and Mk3 [*F*(3,141) = 5.579, *P* = 0.0012] when compared with control monkeys (Figure 2B). Meanwhile, immunohistostaining of TH (+) in the SN showed obvious cell loss, with Mk3 showing the greatest loss and Mk1 the least (Figure 3). Specifically, there was obvious damage in TH (+) neurons, including the reduction of soma size and the intensity of DAB/Ni staining in soma and neurites in the ventral, medial and dorsal SN of MPTP injected monkeys when compared to control monkeys (Figure 3). Quantitative stereology analysis, displayed in Figure 4, demonstrated a significant decrease in areas of TH (+) labeling of all 3 MPTP-injected monkeys when compared with control monkeys [*F*(3,279) = 43.5, *P* < 0.0001] (Figure 4E). However, while the TH (+) labeling was significantly lower in all three MPTP-injected monkeys compared to controls, the labeling of TH (+) in Mk3 was significantly lower than that of Mk2 and Mk1 in an overall analysis combining the entire area of SN [*F*(2,133) = 11.14, *P* < 0.0001] (Figure 4E). There was no significant difference between Mk2 and Mk1 in such analysis (Figure 4E). Similar patterns of DA loss could be seen in sub-areal analysis in the SN tier of A13.4-A12.7 [*F*(2,45) = 16.46, *P* < 0.0001] and A11.6 [*F*(2,30) = 5.075, *P* = 0.0126] (Figures 4B,C). While TH (+) positive area was significantly lower in A15.2-A14 in Mk2 [*F*(3,64) = 4.138, *P* = 0.0096] and Mk3 [*F*(3,62) = 5.902, *P* = 0.0013] when compared to controls (Figures 4A,D), we only observed a reduction in A10.5-A8.4 in Mk1, Mk2, and Mk3.

### The Relationship Between Iron Content and PD Pathology in MPTP-Lesioned Monkeys

**FIGURE 5:**
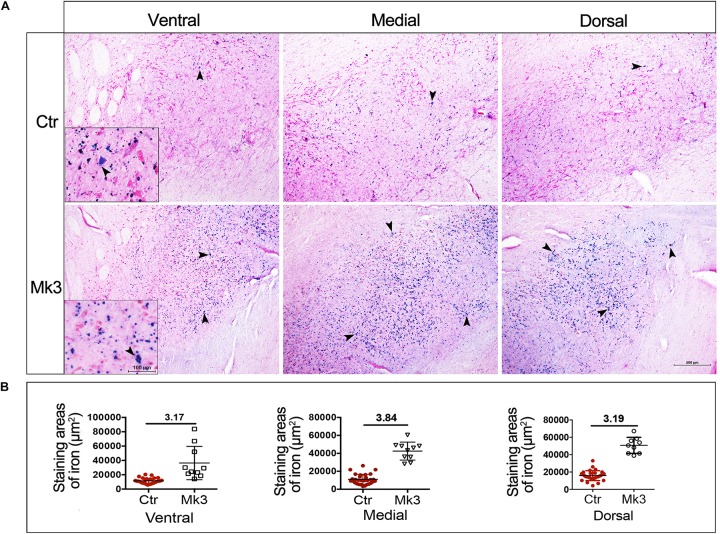
Iron accumulation in the SN in MPTP-lesioned Mk3 monkeys. **(A)** The microphotograph of Perls’ Prussian blue staining (denoting iron) in control and in monkey 3 (Mk3) that was subjected to MPTP injection and displayed the most severe structural and functional deficits among MPTP-lesioned monkeys. Note the obvious increase of blue staining in the SN of Mk3, which can be observed in ventral, medial and dorsal aspects of the SN. The insets (ventral) reveal that the accumulated iron is mainly deposited extracellularly, with relatively little deposition in neuronal somas. **(B)** Quantitative comparison of the area of Perls’ Prussian blue staining in the SN between control monkeys and Mk3. It shows that the areas of staining were obviously elevated in ventral, medial, and dorsal aspects of SN in Mk3 when compared to that of control.

In the present study, accumulation of brown dots was noted in the SN of MPTP-lesioned monkeys (Figure 3, yellow and red arrows, insets). In order to investigate the nature of these dots, we performed the tissue staining in Mk3 using Perls’ Prussian blue which denotes iron. Using such a method, it was obvious that there was significantly more Prussian blue staining in all three regions examined (ventral, medial, and dorsal) in Mk3 compared to the control monkeys (Figure 5). The inset of higher magnification in Figure 5 indicate that, although elevated in both areas, iron content in the extracellular was noticeably higher than in the neuronal somas (Figure 5A). Statistical analysis revealed that the area labeled with Prussian blue in Mk3 was at least threefold greater than that in the control group when examined in ventral, medial, and dorsal SN, respectively (Figure 5B).

**FIGURE 6:**
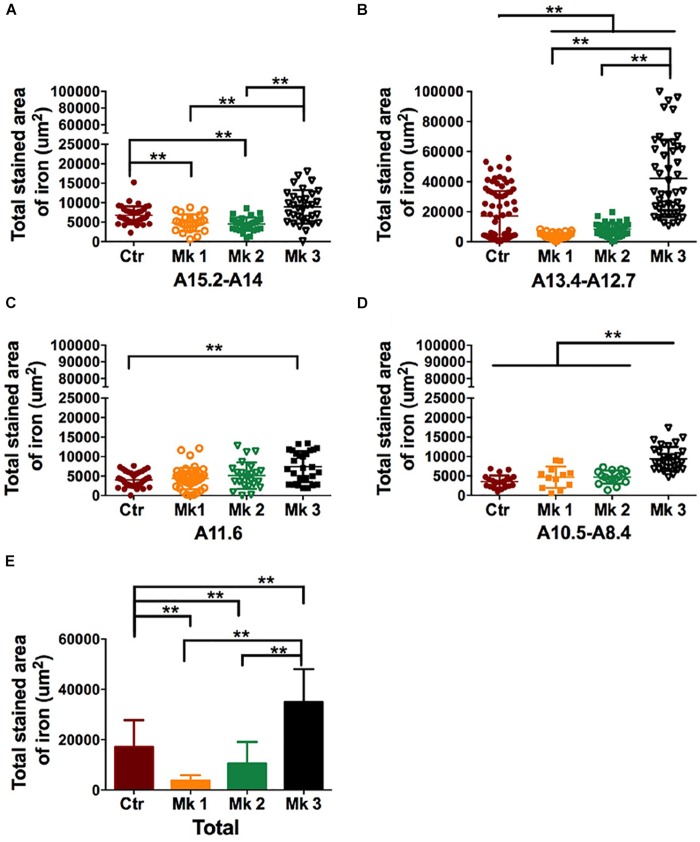
Determination and comparison of iron accumulation in the SN in three MPTP-lesioned monkeys and in controlled monkeys. The data of Perls’ Prussian blue staining was collected and stereology analysis was carried out in four SN tiers for each monkey, A15.2-A14 **(A)**; A13.4-A12.7 **(B)**; A11.6 **(C)**, and A10.5-A8.4 **(D)**. Note that Perls’ Prussian blue staining was consistently elevated in Mk3 compared to control in all tiers. In addition, Perls’ Prussian blue staining in Mk3 was also significantly higher than Mk1 and Mk2 in tiers A15.2-A14 [*F*(2,84) = 20.43, *P* < 0.0001], A13.4-A12.7 [*F*(2,125) = 78.66, *P* < 0.0001] and A10.5-A8.4 [*F*(2,55) = 22.07, *P* < 0.0001]. Interestingly, Perls’ Prussian blue staining was significantly lower in Mk1 and Mk2 in tier A15.2-A14 {Mk1,[*F*(3,127) = 3.996, *P* = 0.0093]; Mk2,[*F*(3,134) = 7.243, *P* = 0.0002]} and A13.4-A12.7 {Mk1, [*F*(3,158) = 16.37, *P* < 0.0001]; Mk2, [*F*(3,162) = 11.33, *P* < 0.0001]} when compared with control. **(E)**: A comparison of Perls’ Prussian blue staining area in each monkey when the data were pooled together from all the SN tiers. Analysis showed that Perls’ Prussian blue staining area in the SN of Mk3 was significantly elevated when compared to control [*F*(3,441) = 18.81, *P* < 0.0001] and to that of Mk1 and 2 [*F*(2,362) = 47.4, *P* < 0.0001]. Interestingly, Perls’ Prussian blue staining areas in Mk1 [*F*(3,427) = 13.04, *P* < 0.0001] and Mk2 [*F*(3,434) = 8.621, *P* < 0.0001] were significantly reduced when compared to control. ANOVA, ^∗^*P* < 0.05, ^∗∗^*P* < 0.01.

To further investigate the relationship between iron accumulation and neuronal degeneration in the SN region, we carried out stereological analysis in all 3 MPTP-injected monkeys (Mk1, Mk2, and Mk3). When analysis was conducted using the data from entire SN, it appears that iron labeling in Mk3 was significantly greater than that in control monkeys [*F*(3,441) = 18.81, *P* < 0.0001] (Figure 6E). Interestingly, there was a reduction of iron labeling in Mk1 [*F*(3,427) = 13.04, *P* < 0.0001] and Mk2 [*F*(3,434) = 8.621, *P* < 0.0001] to a lesser degree, when compared to control (Figure 6E). The same pattern of the change of iron could also be seen in sub-areal analysis in the SN tier A15.2-A14 and A13.4-A12.7 (Figures 6A,B). Therefore, in Mk3, it appears that a significant reduction of TH (+) labeling was associated with a significantly elevated iron staining in the SN (Figures 4–6). This seems to suggest that iron may be a contributing or mediating factor in MPTP-induced DA neuron loss in the SN. However, in Mk1 and Mk2, it appears that, overall, a reduction of TH (+) neuron was accompanied by a reduction of iron (Figures 4, 6).

### Lipofuscin Accumulation in the SN of MPTP-Lesioned Monkeys

**FIGURE 7:**
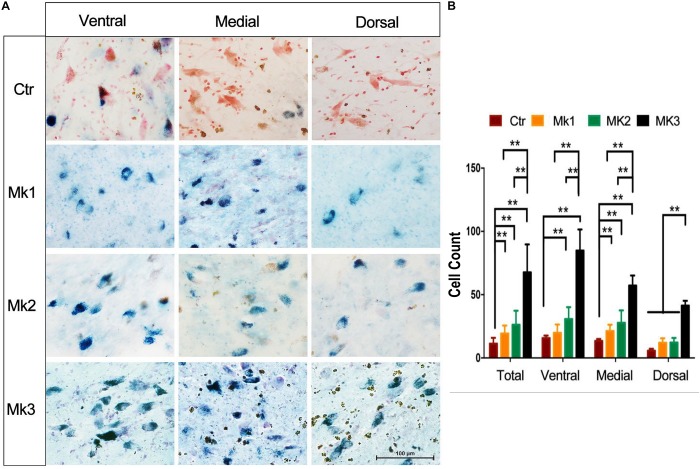
Lipofuscin accumulation in the SN of MPTP-injected monkeys. **(A)** Lipofuscin was accumulated in neurons of the SN in MPTP-injected monkeys. The green or blue staining (Schmorl staining) denote lipofuscin accumulation in neurons, while red indicates control neurons (neutral red). Note that in control monkey, most of the neurons were stained with red, with little sign of green or blue staining. In contrast, MPTP-injected monkeys exhibited a significant amount of neurons with green or blue staining, indicative of lipofuscin accumulation. **(B)** The determination and comparison of the numbers of neurons that contain lipofuscin in control and in MPTP-injected monkeys. Cells that contain lipofuscin in the SN of control and MPTP-injected monkeys were counted and organized in overall (total) and in ventral, medial, and dorsal aspects of the SN. Note that lipofuscin accumulation in Mk3 was consistently the highest among all monkeys in all comparisons [*F*(3,43) = 60.52, *P* < 0.0001]. In addition, lipofuscin accumulation in Mk1 [*F*(3,53) = 10.54, *P* < 0.0001]and Mk2 [*F*(3,56) = 16.12, *P* < 0.0001] was significantly higher than the control in overall comparison and that in medial aspect of the SN [Mk1, *F*(3,20) = 9.408, *P* = 0.0004; Mk2, *F*(3,18) = 7.587, *P* = 0.0017]. Mk2 lipofuscin accumulation was also higher than control in the ventral region [*F*(3,17) = 6.108, *P* = 0.0052]. ANOVA, ^∗∗^*P* < 0.01, ^∗^*P* < 0.05.

Existing studies have found that oxidative stress is likely involved in the MPP^+^-induced DA cell death (Anantharam et al., 2007; Pariyar et al., 2017). In addition, an increase of iron can also lead to the accumulation of lipofuscin, which is an end-product of oxidative attack (Marzabadi et al., 1988; Di Guardo, 2015). We therefore set out to measure the level of lipofuscin as an indicator of oxidative stress. We have found that lipofuscin was significantly accumulated in DA neurons of SN. Specifically, the count of the number of lipofuscin positive cells in MPTP-treated monkeys was significantly greater than that in control animals [*F*(3,94) = 9.295, *P* < 0.0001] (Figure 7A). While such elevation of lipofuscin could be seen in all three monkeys injected with MPTP, Mk3 had the highest elevation [*F*(3,43) = 60.52, *P* < 0.0001] (Figure 7B). No significant difference of the number of lipofuscin positive cells in the SN was noted between Mk2 and Mk1 (Figure 7). Therefore, we again observed the highest elevation of lipofuscin in the SN of Mk3, a monkey that also showed the highest accumulation of iron and the greatest loss of TH (+) neurons.

### Dose-Dependent Effect of Iron Accumulation on Control or MPP^+^ -Exposed Cells *in vitro*

**FIGURE 8:**
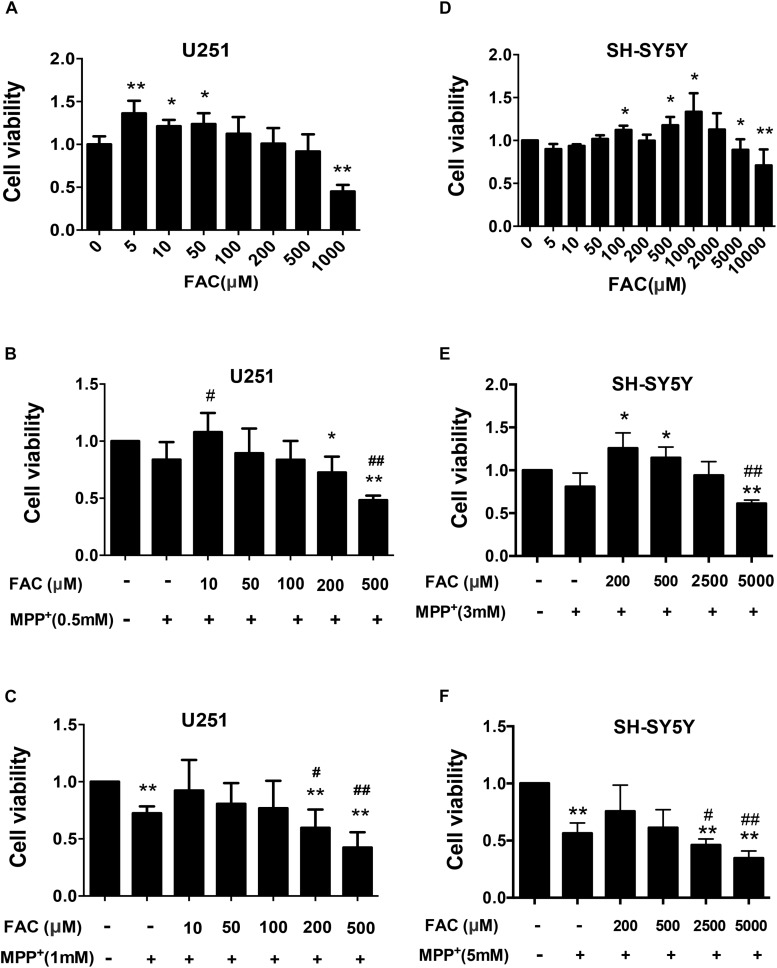
The influence of Ferric ammonium citrate on the cell viability of cultured U251 and SH-SY5Y cells in the presence of MPP^+^. **(A–C)** Cell viability was determined for U251 in the absence **(A)** and in the presence of low **(B)** and high **(C)** levels of MPP^+^. **(D–F)** Cell viability was determined for SH-SY5Y cells in the absence **(D)** and in the presence of low **(E)** and high **(F)** levels of MPP^+^. Note that in the absence of MPP^+^, low levels of ferric ammonium citrate (FAC) enhanced the viability of both cell types, while high levels decreased it **(A,D)**. In the presence of MPP^+^, lower-level FAC either enhanced (when MPP^+^ at lower levels, **B**) or restored (when MPP^+^ high levels, **C**) viability of U251 cells. However, high levels of FAC led to a reduction of cell viability when compared to controls or to either level of MPP^+^ exposure **(B,C)**. Similar to U251 cells, the viability of SH-SY5Y cells in the presence of low levels of MPP^+^ could be enhanced with lower levels of FAC but reduced with high levels of FAC **(E)**. In the presence of high levels of MPP^+^, FAC reduced cell viability **(F)**. Each experiment was repeated four times and all data were expressed as mean + SEM, *t*-test, ^∗^*P* < 0.05, ^∗∗^*P* < 0.01, compared with control; ^#^*P* < 0.05, ^##^*P* < 0.01, compared with MPP^+^ only group.

Subsequently, we investigated iron’s effect on cell viability for control and MPP^+^ exposed cell cultures (U251 and SH-SY5Y). Ferric ammonium citrate (FAC), which has been shown to enhance iron accumulation, was utilized at concentrations consistent with the current relative literature (Li et al., 2016). In the U251 cell (human glioma cells) system, we found that low doses of FAC (5 to 50 μM) increased cell viability. However, high doses of FAC (100 to 500 μM) did not affect the cell viability (Figure 8A). At 1,000 μM, however, FAC induced significant cell death (Figure 8A). Next, we set out to examine the influence of FAC on MPP^+^-induced cell death on U251 cells. In the low level of MPP^+^ (0.5 mM) for which no significant cell death occurred in U251 cells, pretreatment (2 h) with a low dose (10 μM) of FAC led to an increased cell viability in the presence of MPP^+^ (Figure 8B). At higher doses (200 and 500 μM) of FAC, however, cell viability was significantly reduced (Figure 8B). This indicates that while MPP^+^ at 0.5 mM, or FAC at 200 and 500 μM was not toxic when applied separately, a combination of these two was toxic (Figures 8A,B). At a higher level of MPP^+^ (1 mM) for which cell death did occur, lower level of FAC (10, 50, and 100 μM) could reverse the cell death caused by MPP^+^ (Figure 8C). However, higher levels of FAC (200 and 500 μM) significantly exacerbated the cell death caused by MPP^+^ alone (Figure 8C). Similar results were also obtained from the SH-SY5Y cell system (Figures 8D–F). Specifically, when applied alone, FAC at low concentration (100, 500, and 1,000 μM) could increase cell viability, while at high concentrations (5,000 and 10,000 μM), FAC could reduce cell viability (Figure 8D). Similar to that of U251 cells, in the presence of low MPP^+^ (3 mM) that was non-toxic, low level of FAC (200 and 500 μM) increased cell viability, while high level of FAC (5,000 μM) lowered the cell viability when compared to control or MPP^+^ only (Figure 8E). However, in the presence of higher concentrations of MPP^+^ (5 mM), which caused significant levels of cell death in SH-SY5Y cells, additional higher levels of FAC (2,500 and 5,000 μM) exacerbated the cell death caused by MPP^+^ alone (Figure 8F).

## Discussion

Previous studies have demonstrated the abnormal iron accumulation in the SN of PD patients (Vymazal et al., 1999; Ke and Qian, 2007) and that high intake of iron in diet was associated with an increased risk of PD (Powers et al., 2003). Further, studies show that excessive iron content may be related to clinical symptoms (He et al., 2013; Guan et al., 2017; Naduthota et al., 2017) and contributes to DA neuron degeneration in PD (Ke and Ming Qian, 2003; Oakley et al., 2007; Ward et al., 2014). In the current study, we have observed that as one of the three monkeys subjected to MTPT-exposure, Mk3 displayed the most severe clinical motor deficits among all the monkeys, control or MPTP-injected. Importantly, Mk3 was also associated with the highest level of iron accumulation, oxidative stress, and DA cell loss in the SN, suggesting a correlation of heightened iron accumulation with severe PD-like motor deficits. In good agreement with the *in vivo* studies, we have also shown that the addition of FAC, a known compound promoting iron accumulation, exacerbated MPTP toxicity in cultured U251 and SH-SY5Y cells when used in high concentrations. Therefore, our data are consistent with the growing evidence suggesting that significantly high level of iron is associated with, and likely a contributing factor in, DA cell loss and behavioral deficits in PD.

Iron-induced oxidative stress has been widely implicated in the pathogenesis of neurodegenerative diseases, including PD. Excessive iron can participate in Fenton’s reaction to generate ROS (Gaasch et al., 2007), which has the potential to damage DNA, modify proteins leading to structural and functional alterations (Dalle-Donne et al., 2003a, b; Melis et al., 2013), and promote peroxidation of polyunsaturated fatty acids leading to alterations and functional loss of membranes (Catala, 2009). Furthermore, as a well-established pathology in PD, oxidative stress and therefore ROS can stimulate the release of iron from its storage proteins such as Fe-S cluster proteins and further feed ROS via Fenton’s reaction (Zucca et al., 2017). Therefore, high brain levels of iron content may not only contribute to, but also result from neurodegeneration. While the exact role of iron in PD neurodegeneration is still not clear, it is likely that the interplay of iron and oxidative stress is involved in a feedforward vicious cycle of continuously illicit cellular damage that leads to death of DA neurons in PD.

In the present study, we also noticed that, compared to neuronal soma, the extracellular iron content was conspicuously high. As such, in addition to neuronal soma, neurites may also be directly exposed to a high level of iron which could make neuronal processes more vulnerable to oxidative stress and then likely be damaged in the event of MPTP injection. This neuronal process degeneration could then progress to the failure of dopamine transmission from SN to striatum and consequently to the accumulation of dopamine in DA neurons in SN. Normally, excess dopamine in DA neurons can be removed by neuromelanin (NM), and then dopamine could be converted into a stable compound, clamping toxicities in the DA cells (Zucca et al., 2017). The major iron-protein complex in DA neurons is the neuromelanin-iron complex, since NM is an effective metal chelator, trapping iron and providing neuronal protection from oxidative stress (Zucca et al., 2017). However, the free iron will elevate if NM reaches its maximal capacity of chelating iron in the event of excessive iron. Consequently, intracellular dopamine will be oxidized by iron to DA-*o*-quinone, an important electrophilic reactive molecule with known toxicity. DA-*o*-quinone has been shown to form adducts with amino acid residues (mainly cysteine residues) of different proteins (Hare and Double, 2016; Zucca et al., 2017). Specifically, DA-*o*-quinone has been demonstrated to promote aggregation of α-synuclein, a hallmark of neuropathology in PD (Lotharius and Brundin, 2002; Yamakawa et al., 2010). In fact, the protein sequence of α-syn has a remarkable amount of lysine that is known to be easily acetylated by aldehydes (Plotegher and Bubacco, 2016), leading to the alteration of the membrane-bound α-helical structure and the aggregation of α-syn. In addition to DA-*o*-quinone, 6-OHDA, a neurotoxin commonly used in animal models of PD, is also a minor byproduct of iron-mediated dopamine oxidation, which can inhibit mitochondrial complexes I and IV and reduce ATP production, leading to eventual cell death (Hare and Double, 2016). Additionally, a recent study found that exposure of macrophages to dopamine has been shown to enhance the uptake of non-transferrin bound iron into cells (Dichtl et al., 2018). The result indicates that the accumulated DA may increase cellular iron content which then leads to oxidative stress response. Taken together, iron has been shown to be associated with a variety of pathological mechanisms leading to DA neuron degeneration.

In addition to encouraging α-syn aggregation though the pathway of DA-*o*-quinone, iron may stimulate α-synuclein aggregation through direct binding. For example, Friedlich et al. (2007) have shown that sequence homology to iron responsive elements (IRE) exist in the α-syn mRNA 5′-untranslated region, suggesting direct binding of iron and α-syn. Some other studies and our previous work that upregulated α-syn found in MPTP group monkeys (Shi et al., 2014) demonstrated that iron can induce α-syn aggregation, oligomerization or even formation of fibrils both in cell and free-cell systems (Ostrerova-Golts et al., 2000; Bharathi et al., 2007; He et al., 2013), further solidifying the notion of that iron can directly bind to α-syn and cause its aggregation. Interestingly, Baksi et al. (2016) found that impaired or altered function of α-syn can cause iron dyshomeostasis in retinal cells. Therefore, similar to oxidative stress and iron, α-syn and iron may also interact with each other to promote α-syn aggregation and thereby the progression of PD.

**FIGURE 9:**
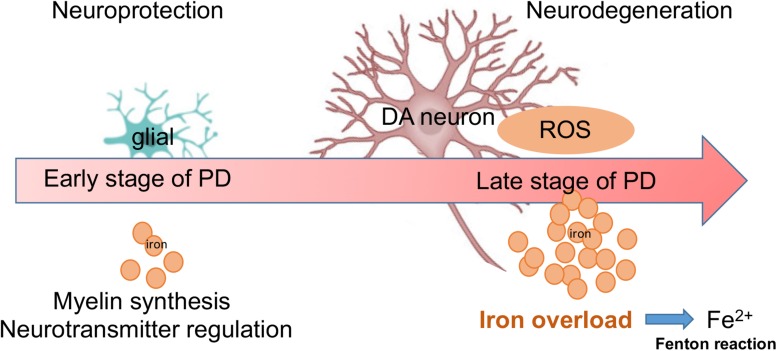
A summary diagram is shown. Both glial and DA neurons are involved with iron metabolism in the progression of PD. During early stage of PD, low dose of iron may offer neuroprotection via myelin synthesis and neurotransmitter regulation. However, during later stages of PD, increased levels of iron may cause neurodegeneration through large amounts of ROS (Fenton reaction).

Despite strong evidence of iron neurotoxicity in PD, the function of iron is still far from clear. For example, based on our monkeys and cell culture investigation in the current study, the effect of iron on cell viability related to PD pathology does not appear to be detrimental in all cases. For example, compared to Mk3, Mk1 and Mk2 displayed significantly less severe motor deficits and neuronal cellular damage associated with PD pathologies. Interestingly, the overall iron content in the SN region of both Mk1 and Mk2 was decreased compared to that of control monkeys. This seems to suggest that iron levels in the SN of monkeys with PD may consist of two phases: an early, less severe phase, where iron levels are depressed, and a later, more severe phase with elevated levels of iron. Although the pathological significance of elevated iron levels in relation to neurodegeneration is supported by most of the existing studies, the significance of early stage iron depression is not clear. Regardless, as the PD pathology advances, iron levels appear to eventually elevate, which is likely due to the overwhelming oxidative stress and degenerative process (Figure 9).

It is well established that iron is an essential metal that is critical for cellular energy metabolism in physiological processes, such as glucose metabolism, the Krebs cycle, and oxidative phosphorylation (Oexle et al., 1999). Therefore, it is possible that the high demand for energy (and therefore iron) during the early stages of PD led to the decreased iron levels, as seen in Mk1 and Mk2. This could be viewed as a sign of stress during the early stages of neurodegeneration. In this period, the DA neurons, although facing an initial PD related pathological attack, continue to initiate or sustain their repair processes by consuming additional energy (and iron). Consistent with such observations, Ayton et al. (2015) found that the iron content in the SN of mice during early stages (3 days after MPTP injection) was reduced. However, this reduction could have also possibly been caused by the significantly increased levels of APP. Therefore, while we report reduced iron levels in the earlier stages of MPTP induced PD in this investigation, it is obvious that the significance of these changes is yet to be determined.

These *in vivo* studies are in good agreement with our parallel *in vitro* investigation. In the cell culture study, we noted that mild and moderate levels of FAC, a compound that enhances iron accumulation, when applied either alone or in the presence of MPP^+^ (FAC applied before the exposure of MPP^+^), did not cause or exacerbate cell death instigated by MPP^+^. However, at high concentrations, FAC increased cell death with or without MPP^+^. It is also clear that at higher levels iron can be toxic, even in the absence of or at low levels of MPP^+^, which is likely due to, at least in part, the stimulation of oxidative stress (a well-known pathology).

Lipofuscin is a known end product of oxidative stress (Thaw et al., 1984). It has been found that lipofuscin accumulation can be accelerated by increased oxidative stress in cultured rat neonatal myocardial cells (Marzabadi et al., 1991), while iron could increase the level of lipofuscin accumulation in the culture medium (Marzabadi et al., 1988). This indicates that both iron and oxidative stress promote lipofuscin accumulation. In the present study, we found that lipofuscin accumulation occurred in the SN, and the levels of accumulation were positively correlated to iron contents, DA neurons loss, and PD scores. Specifically, the level of lipofuscin was the highest in Mk3, which was also associated with the highest level of neurodegeneration and motor deficits. This data is consistent with several previous studies that MPTP accelerated the accumulation of lipofuscin in mouse adrenal gland (Hadjiconstantinou et al., 1987) and retina (Mariani et al., 1986). Taken together, our data, coupled with others’, suggest that MPTP-induced oxidative stress, indicated by lipofuscin, can likely lead to the accumulation of iron, which in turn, in a feed forward loop, exacerbates MPTP toxicity and leads to cell loss of DA neurons in PD.

Following the demonstrating of the well-established iron toxicity in PD, iron chelation therapy in PD has been examined as a novel treatment option, first in animal models and recently in human studies. For example, clinical studies have shown that deferiprone (DFO), an iron chelator, could reduce iron content in the dentate and caudate nucleus (Lilley, 2012; Martin-Bastida et al., 2017) and provide neuroprotection (Aguirre et al., 2015). These studies not only further solidify the toxic role of iron in PD, but also suggest novel therapy of iron chelation. However, in light of our own current findings, iron chelating therapy may be suitable in more severe or later stages of PD which are associated with high iron contents, but not appropriate in less severe or early stages of PD pathology with depressed iron level.

## Conclusion

In a monkey model of PD using MPTP, we have gathered findings that support the notion that excessive iron is likely a causal factor mediating and accelerating MPTP/MPP^+^ toxicity leading to DA neuron cell death. The exact mechanism by which iron together with oxidative stress mediate the MPTP/MPP^+^-induced degeneration that leads to the SN specific neuron loss is not clear. However, the current results indicate that the interplay of excessive iron with neuromelanin, DA, α-syn, and the process of oxidative may be a key factor in causing cell death of DA neurons in the SN. Therefore, it is likely that excessive iron may increase the risk of PD in humans, along with various other identified environmental risk factors. Meanwhile, our *in vivo* and *in vitro* data also indicate that iron is suppressed during the early stages of PD, however its significance remains unclear and will be the topic of a future study. As such, any possible treatment strategies for influencing iron levels in PD may need to take the timing of treatment into consideration, likely by reducing iron during the late stages of PD when iron is high in the SN (to suppress degeneration).

## Data Availability

All datasets generated for this study are included in the manuscript and/or the Supplementary Files.

## Ethics Statement

All the experimental protocols had been reviewed and approved by the Animal Welfare and Use Committee, and all the experimental procedures were in conformity with the guidance of “the National Institutes of Health Guide for the Care and Use of Laboratory Animal” of the United States.

## Author Contributions

ZC and RS designed the overall study and contributed to the writing of the manuscript. LS performed all of the experiments related to clinical motor evaluations, immunohistochemical analysis, data analysis, and contributed to the writing of the manuscript. ER assisted with statistical analysis, data analysis, figure preparations, and contributed to the writing of the manuscript. WM performed the experiments related to animal feeding and management, tissue collection and histopathology. CH and QL contributed to planning some of the experiments and contributed to revising the manuscript. WZ, LG, and AC provided the funds and reagents. WL, YX, JF, and LT coordinated and supervised the project. All authors approved the final version of the manuscript.

## Conflict of Interest Statement

The authors declare that the research was conducted in the absence of any commercial or financial relationships that could be construed as a potential conflict of interest.

## Supplementary Material

FIGURE S1 Semi-diagrammatic illustrations of the SN of macaque monkey brain in stereotaxic coordinates from frontal planes A15.2-A8.4. The nucleus of the nigra was labeled with red circle. SNc, substantia nigra pars compact; SNd, substantia nigra, pars diffusa; St, nucleus subthalamicus.

## References

[B1] Agid Y. (1991). Parkinson’s disease: pathophysiology. *Lancet* 337 1321–1324. 1674304 10.1016/0140-6736(91)92989-f

[B2] Aguirre P. Mena N. P. Carrasco C. M. Munoz Y. Perez-Henriquez P. Morales R. A. (2015). iron chelators and antioxidants regenerate neuritic tree and nigrostriatal fibers of MPP+/MPTP-lesioned dopaminergic neurons. *PLoS One* 10:e0144848. 10.1371/journal.pone.0144848 26658949 PMC4684383

[B3] An H. Zeng X. Niu T. Li G. Yang J. Zheng L. (2018). Quantifying iron deposition within the substantia nigra of Parkinson’s disease by quantitative susceptibility mapping. *J. Neurol. Sci.* 386 46–52. 10.1016/j.jns.2018.01.008 29406966

[B4] Anantharam V. Kaul S. Song C. Kanthasamy A. Kanthasamy A. G. (2007). Pharmacological inhibition of neuronal NADPH oxidase protects against 1-methyl-4-phenylpyridinium (MPP+)-induced oxidative stress and apoptosis in mesencephalic dopaminergic neuronal cells. *Neurotoxicology* 28 988–997. 10.1016/j.neuro.2007.08.008 17904225 PMC2140261

[B5] Ayton S. Lei P. Hare D. J. Duce J. A. George J. L. Adlard P. A. (2015). Parkinson’s disease iron deposition caused by nitric oxide-induced loss of beta-amyloid precursor protein. *J. Neurosci.* 35 3591–3597. 10.1523/JNEUROSCI.3439-14.2015 25716857 PMC6605561

[B6] Baksi S. Tripathi A. K. Singh N. (2016). Alpha-synuclein modulates retinal iron homeostasis by facilitating the uptake of transferrin-bound iron: implications for visual manifestations of Parkinson’s disease. *Free Radic. Biol. Med.* 97 292–306. 10.1016/j.freeradbiomed.2016.06.025 27343690 PMC4996775

[B7] Ben-Shachar D. Youdim M. B. (1991). Intranigral iron injection induces behavioral and biochemical “parkinsonism” in rats. *J. Neurochem.* 57 2133–2135. 10.1111/j.1471-4159.1991.tb06432.x 1940919

[B8] Bharathi, Indi S. S. Rao K. S. (2007). Copper- and iron-induced differential fibril formation in alpha-synuclein: TEM study. *Neurosci. Lett.* 424 78–82. 10.1016/j.neulet.2007.06.052 17714865

[B9] Catala A. (2009). Lipid peroxidation of membrane phospholipids generates hydroxy-alkenals and oxidized phospholipids active in physiological and/or pathological conditions. *Chem. Phys. Lipids* 157 1–11. 10.1016/j.chemphyslip.2008.09.004 18977338

[B10] Chen Z. L. Fan G. L. Luo Q. H. Zhu C. M. Huang Y. D. (2007). Effects of estrogen on er, ngf, and chat expression in cerebellum of aging female sprague-dawley rat. *Agric.; Sci. China* 6 368–374. 10.1016/s1671-2927(07)60058-3

[B11] Dalle-Donne I. Giustarini D. Colombo R. Rossi R. Milzani A. (2003a). Protein carbonylation in human diseases. *Trends Mol. Med.* 9 169–176. 10.1016/s1471-4914(03)00031-5 12727143

[B12] Dalle-Donne I. Rossi R. Giustarini D. Milzani A. Colombo R. (2003b). Protein carbonyl groups as biomarkers of oxidative stress. *Clin. Chim. Acta* 329 23–38. 10.1016/s0009-8981(03)00003-2 12589963

[B13] Deng X. Liang Y. Lu H. Yang Z. Liu R. Wang J. (2013). Co-transplantation of GDNF-overexpressing neural stem cells and fetal dopaminergic neurons mitigates motor symptoms in a rat model of Parkinson’s disease. *PLoS One* 8:e80880. 10.1371/journal.pone.0080880 24312503 PMC3849044

[B14] Dexter D. T. Wells F. R. Lees A. J. Agid F. Agid Y. Jenner P. (1989). Increased nigral iron content and alterations in other metal ions occurring in brain in Parkinson’s disease. *J. Neurochem.* 52 1830–1836. 10.1111/j.1471-4159.1989.tb07264.x 2723638

[B15] Di Guardo G. (2015). Lipofuscin, lipofuscin-like pigments and autofluorescence. *Eur. J. Histochem.* 59:2485. 10.4081/ejh.2015.2485 25820564 PMC4378218

[B16] Dichtl S. Haschka D. Nairz M. Seifert M. Volani C. Lutz O. (2018). Dopamine promotes cellular iron accumulation and oxidative stress responses in macrophages. *Biochem. Pharmacol.* 148 193–201. 10.1016/j.bcp.2017.12.001 29208364

[B17] Foley P. Riederer P. (2000). Influence of neurotoxins and oxidative stress on the onset and progression of Parkinson’s disease. *J. Neurol.* 247(Suppl. 2), II82–II94. 10991671 10.1007/pl00007766

[B18] Fox S. H. Johnston T. H. Li Q. Brotchie J. Bezard E. (2012). A critique of available scales and presentation of the non-human primate dyskinesia rating scale. *Mov. Disord.* 27 1373–1378. 10.1002/mds.25133 22976821

[B19] Friedlich A. L. Tanzi R. E. Rogers J. T. (2007). The 5’-untranslated region of Parkinson’s disease alpha-synuclein messengerRNA contains a predicted iron responsive element. *Mol. Psychiatry* 12 222–223. 10.1038/sj.mp.4001937 17325711

[B20] Gaasch J. A. Lockman P. R. Geldenhuys W. J. Allen D. D. Van Der Schyf C. J. (2007). Brain iron toxicity: differential responses of astrocytes, neurons, and endothelial cells. *Neurochem. Res.* 32 1196–1208. 10.1007/s11064-007-9290-4 17404839

[B21] Glaser E. M. Van der Loos H. (1981). Analysis of thick brain sections by obverse-reverse computer microscopy: application of a new, high clarity golgi-nissl stain. *J. Neurosci. Methods* 4 117–125. 10.1016/0165-0270(81)90045-5 6168870

[B22] Goldfischer S. Bernstein J. (1969). Lipofuscin (aging) pigment granules of the newborn human liver. *J. Cell Biol.* 42 253–261. 10.1083/jcb.42.1.253 4182373 PMC2107576

[B23] Guan X. Xuan M. Gu Q. Xu X. Huang P. Wang N. (2017). Influence of regional iron on the motor impairments of Parkinson’s disease: a quantitative susceptibility mapping study. *J. Magn. Reson. Imaging* 45 1335–1342. 10.1002/jmri.25434 27545971

[B24] Hadjiconstantinou M. Tjioe S. Alho H. Miller C. Neff N. H. (1987). 1-Methyl-4-phenyl-1,2,3,6-tetrahydropyridine (MPTP) accelerates the accumulation of lipofuscin in mouse adrenal gland. *Neurosci. Lett.* 83 1–6. 10.1016/0304-3940(87)90206-0 3502018

[B25] Hare D. J. Double K. L. (2016). Iron and dopamine: a toxic couple. *Brain* 139 1026–1035. 10.1093/brain/aww022 26962053

[B26] He Q. Song N. Jia F. Xu H. Yu X. Xie J. (2013). Role of alpha-synuclein aggregation and the nuclear factor E2-related factor 2/heme oxygenase-1 pathway in iron-induced neurotoxicity. *Int. J. Biochem. Cell Biol.* 45 1019–1030. 10.1016/j.biocel.2013.02.012 23454680

[B27] He Y. Thong P. S. Lee T. Leong S. K. Mao B. Y. Dong F. (2003). Dopaminergic cell death precedes iron elevation in MPTP-injected monkeys. *Free Radic. Biol. Med.* 35 540–547. 10.1016/s0891-5849(03)00385-x 12927603

[B28] Ke Y. Ming Qian Z. (2003). Iron misregulation in the brain: a primary cause of neurodegenerative disorders. *Lancet Neurol.* 2 246–253. 10.1016/s1474-4422(03)00353-3 12849213

[B29] Ke Y. Qian Z. M. (2007). Brain iron metabolism: neurobiology and neurochemistry. *Prog. Neurobiol.* 83 149–173. 10.1016/j.pneurobio.2007.07.009 17870230

[B30] Li S. W. Liu C. M. Guo J. Marcondes A. M. Deeg J. Li X. (2016). Iron overload induced by ferric ammonium citrate triggers reactive oxygen species-mediated apoptosis via both extrinsic and intrinsic pathways in human hepatic cells. *Hum. Exp. Toxicol.* 35 598–607. 10.1177/0960327115597312 26224043

[B31] Lilley R. K. (2012). *ClinicalTrials.gov [Internet]. Identifier NCT00943748, Efficacy and Safety of the Iron Chelator Deferiprone in Parkinson’s Disease (FAIR-PARK-I)*. Available at: https://clinicaltrials.gov/ct2/show/NCT00943748

[B32] Lotharius J. Brundin P. (2002). Pathogenesis of Parkinson’s disease: dopamine, vesicles and alpha-synuclein. *Nat. Rev. Neurosci.* 3 932–942. 10.1038/nrn983 12461550

[B33] Mariani A. P. Neff N. H. Hadjiconstantinou M. (1986). 1-Methyl-4-phenyl-1,2,3,6-tetrahydropyridine (MPTP) treatment decreases dopamine and increases lipofuscin in mouse retina. *Neurosci. Lett.* 72 221–226. 10.1016/0304-3940(86)90084-4 3492691

[B34] Martin-Bastida A. Lao-Kaim N. P. Loane C. Politis M. Roussakis A. A. Valle-Guzman N. (2017). Motor associations of iron accumulation in deep grey matter nuclei in Parkinson’s disease: a cross-sectional study of iron-related magnetic resonance imaging susceptibility. *Eur. J. Neurol.* 24 357–365. 10.1111/ene.13208 27982501

[B35] Marzabadi M. R. Sohal R. S. Brunk U. T. (1988). Effect of ferric iron and desferrioxamine on lipofuscin accumulation in cultured rat heart myocytes. *Mech. Ageing Dev.* 46 145–157. 10.1016/0047-6374(88)90122-4 3226156

[B36] Marzabadi M. R. Sohal R. S. Brunk U. T. (1991). Mechanisms of lipofuscinogenesis: effect of the inhibition of lysosomal proteinases and lipases under varying concentrations of ambient oxygen in cultured rat neonatal myocardial cells. *APMIS* 99 416–426. 10.1111/j.1699-0463.1991.tb05170.x 2043354

[B37] Melis J. P. Van Steeg H. Luijten M. (2013). Oxidative DNA damage and nucleotide excision repair. *Antioxid. Redox Signal.* 18 2409–2419. 10.1089/ars.2012.5036 23216312 PMC3671630

[B38] Mochizuki H. Imai H. Endo K. Yokomizo K. Murata Y. Hattori N. (1994). Iron accumulation in the substantia nigra of 1-methyl-4-phenyl-1,2,3,6-tetrahydropyridine (MPTP)-induced hemiparkinsonian monkeys. *Neurosci. Lett.* 168 251–253. 10.1016/0304-3940(94)90462-6 8028787

[B39] Moos T. Rosengren Nielsen T. Skjorringe T. Morgan E. H. (2007). Iron trafficking inside the brain. *J. Neurochem.* 103 1730–1740. 10.1111/j.1471-4159.2007.04976.x 17953660

[B40] Naduthota R. M. Honnedevasthana A. A. Lenka A. Saini J. Geethanath S. Bharath R. D. (2017). Association of freezing of gait with nigral iron accumulation in patients with Parkinson’s disease. *J. Neurol. Sci.* 382 61–65. 10.1016/j.jns.2017.09.033 29111022

[B41] Oakley A. E. Collingwood J. F. Dobson J. Love G. Perrott H. R. Edwardson J. A. (2007). Individual dopaminergic neurons show raised iron levels in Parkinson disease. *Neurology* 68 1820–1825. 10.1212/01.wnl.0000262033.01945.9a 17515544

[B42] Oexle H. Gnaiger E. Weiss G. (1999). Iron-dependent changes in cellular energy metabolism: influence on citric acid cycle and oxidative phosphorylation. *Biochim. Biophys. Acta* 1413 99–107. 10.1016/s0005-2728(99)00088-2 10556622

[B43] Ostrerova-Golts N. Petrucelli L. Hardy J. Lee J. M. Farer M. Wolozin B. (2000). The A53T alpha-synuclein mutation increases iron-dependent aggregation and toxicity. *J. Neurosci.* 20 6048–6054. 10.1523/jneurosci.20-16-06048.2000 10934254 PMC6772599

[B44] Pariyar R. Lamichhane R. Jung H. J. Kim S. Y. Seo J. (2017). Sulfuretin attenuates MPP(+)-induced neurotoxicity through Akt/GSK3beta and ERK signaling pathways. *Int. J. Mol. Sci.* 18 E2753. 10.3390/ijms18122753 29257079 PMC5751352

[B45] Plotegher N. Bubacco L. (2016). Lysines, achilles’ heel in alpha-synuclein conversion to a deadly neuronal endotoxin. *Ageing Res. Rev.* 26 62–71. 10.1016/j.arr.2015.12.002 26690800

[B46] Powers K. M. Smith-Weller T. Franklin G. M. Longstreth WT Jr Swanson P. D. Checkoway H. (2003). Parkinson’s disease risks associated with dietary iron, manganese, and other nutrient intakes. *Neurology* 60 1761–1766. 10.1212/01.wnl.0000068021.13945.7f 12796527

[B47] Riederer P. Wuketich S. (1976). Time course of nigrostriatal degeneration in parkinson’s disease. A detailed study of influential factors in human brain amine analysis. *J. Neural. Transm.* 38 277–301. 10.1007/bf01249445 956814

[B48] Sengstock G. J. Olanow C. W. Menzies R. A. Dunn A. J. Arendash G. W. (1993). Infusion of iron into the rat substantia nigra: nigral pathology and dose-dependent loss of striatal dopaminergic markers. *J. Neurosci. Res.* 35 67–82. 10.1002/jnr.490350109 7685399

[B49] Sharma N. Nehru B. (2018). Curcumin affords neuroprotection and inhibits alpha-synuclein aggregation in lipopolysaccharide-induced Parkinson’s disease model. *Inflammopharmacology* 26 349–360. 10.1007/s10787-017-0402-8 29027056

[B50] Shi L. Q. Luo Q. H. Zeng W. Gong L. Cheng A. C. Bi F. J. (2014). Preliminary establishment of chronic. Parkinson’s disease in rhesus monkey model induced by injection of MPTP. *J. Zhejiang Univ.* 40 257–265.

[B51] Sundberg R. D. Broman H. (1955). The application of the Prussian blue stain to previously stained films of blood and bone marrow. *Blood* 10 160–166. 13230168

[B52] Szabo J. Cowan W. M. (1984). A stereotaxic atlas of the brain of the cynomolgus monkey (*Macaca fascicularis*). *J. Comp. Neurol.* 222 265–300. 10.1002/cne.902220208 6365984

[B53] Tanji K. Imaizumi T. Yoshida H. Mori F. Yoshimoto M. Satoh K. (2001). Expression of alpha-synuclein in a human glioma cell line and its up-regulation by interleukin-1beta. *Neuroreport* 12 1909–1912. 10.1097/00001756-200107030-00028 11435921

[B54] Thaw H. H. Collins V. P. Brunk U. T. (1984). Influence of oxygen tension, pro-oxidants and antioxidants on the formation of lipid peroxidation products (lipofuscin) in individual cultivated human glial cells. *Mech. Ageing Dev.* 24 211–223. 10.1016/0047-6374(84)90072-1 6717089

[B55] Todorich B. Pasquini J. M. Garcia C. I. Paez P. M. Connor J. R. (2009). Oligodendrocytes and myelination: the role of iron. *Glia* 57 467–478. 10.1002/glia.20784 18837051

[B56] van der Stelt M. Fox S. H. Hill M. Crossman A. R. Petrosino S. Di Marzo V. (2005). A role for endocannabinoids in the generation of parkinsonism and levodopa-induced dyskinesia in MPTP-lesioned non-human primate models of Parkinson’s disease. *FASEB J.* 19 1140–1142. 10.1096/fj.04-3010fje 15894565

[B57] Vymazal J. Righini A. Brooks R. A. Canesi M. Mariani C. Leonardi M. (1999). T1 and T2 in the brain of healthy subjects, patients with Parkinson disease, and patients with multiple system atrophy: relation to iron content. *Radiology* 211 489–495. 10.1148/radiology.211.2.r99ma53489 10228533

[B58] Ward R. J. Zucca F. A. Duyn J. H. Crichton R. R. Zecca L. (2014). The role of iron in brain ageing and neurodegenerative disorders. *Lancet Neurol.* 13 1045–1060. 10.1016/s1474-4422(14)70117-6 25231526 PMC5672917

[B59] Xicoy H. Wieringa B. Martens G. J. (2017). The SH-SY5Y cell line in Parkinson’s disease research: a systematic review. *Mol. Neurodegener.* 12:10. 10.1186/s13024-017-0149-0 28118852 PMC5259880

[B60] Yamakawa K. Izumi Y. Takeuchi H. Yamamoto N. Kume T. Akaike A. (2010). Dopamine facilitates alpha-synuclein oligomerization in human neuroblastoma SH-SY5Y cells. *Biochem. Biophys. Res. Commun.* 391 129–134. 10.1016/j.bbrc.2009.11.015 19900407

[B61] Yang W. G. Liu J. Luo Y. Q. Chen P. L. Zhang C. R. Wan X. C. (1990). *A Stereotaxic Atlas of the Brain of Tupaia Belangeri and Macaque Monkey Living in Guangxi.* Guangxi. China: Guangxi Science & Technology Publishing House.

[B62] You L. H. Li F. Wang L. Zhao S. E. Wang S. M. Zhang L. L. (2015). Brain iron accumulation exacerbates the pathogenesis of MPTP-induced Parkinson’s disease. *Neuroscience* 284 234–246. 10.1016/j.neuroscience.2014.09.071 25301748

[B63] Zecca L. Youdim M. B. Riederer P. Connor J. R. Crichton R. R. (2004). Iron, brain ageing and neurodegenerative disorders. *Nat. Rev. Neurosci.* 5 863–873. 10.1038/nrn1537 15496864

[B64] Zucca F. A. Segura-Aguilar J. Ferrari E. Munoz P. Paris I. Sulzer D. (2017). Interactions of iron, dopamine and neuromelanin pathways in brain aging and Parkinson’s disease. *Prog. Neurobiol.* 155 96–119. 10.1016/j.pneurobio.2015.09.012 26455458 PMC4826627

