# Supplementary figures and images for: The Association of Iron and the Pathologies of Parkinson’s Diseases in MPTP/MPP+-Induced Neuronal Degeneration in Non-human Primates and in Cell Culture
Source: Front Aging Neurosci. 2019 Aug 30;11:215. doi: 10.3389/fnagi.2019.00215 (PMC6729105; doi:10.3389/fnagi.2019.00215)

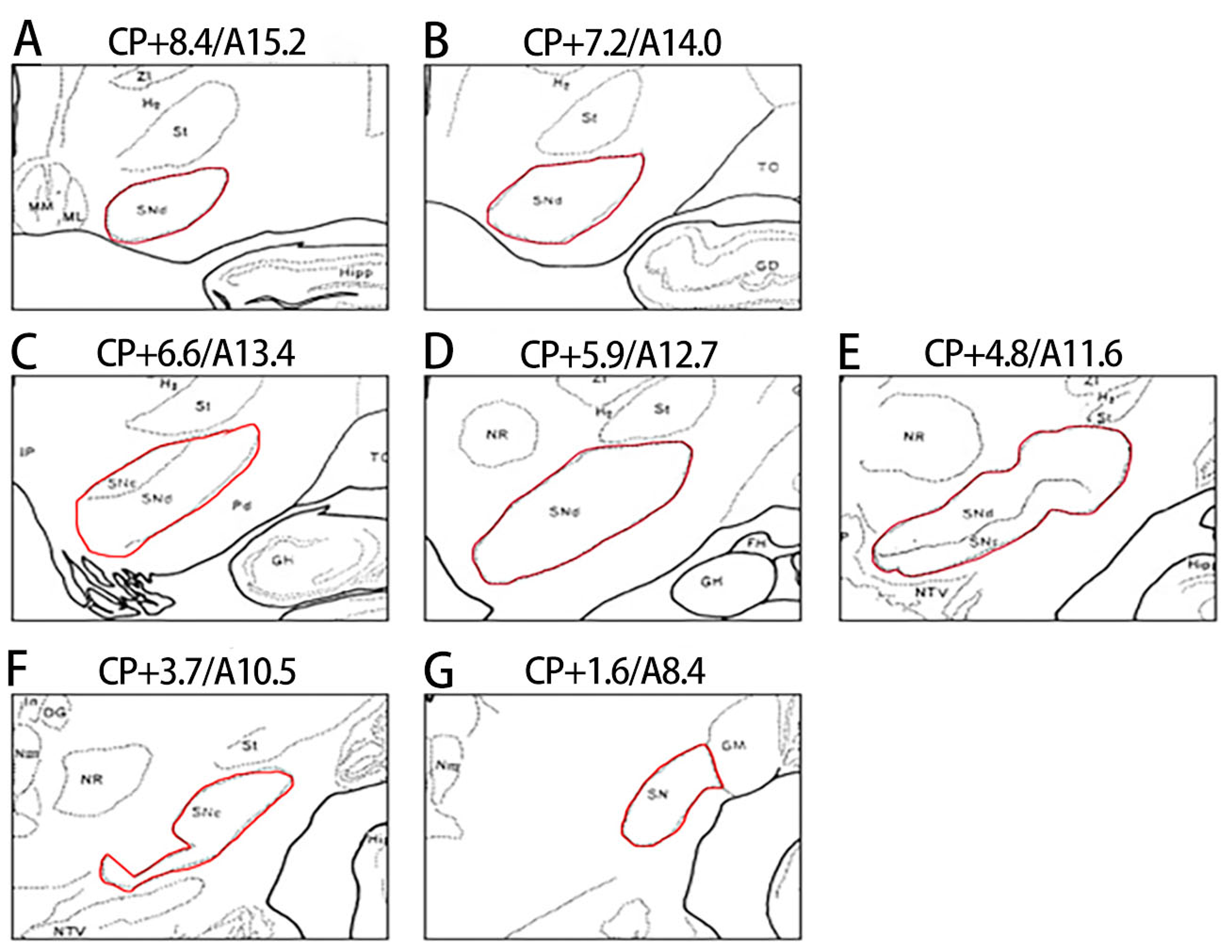

Supplement: FIGURE S1 — Semi-diagrammatic illustrations of the SN of macaque monkey brain in stereotaxic coordinates from frontal planes A15.2-A8.4. The nucleus of the nigra was labeled with red circle. SNc, substantia nigra pars compact; SNd, substantia nigra, pars diffusa; St, nucleus subthalamicus. [file Image_1.tif]
